# Effectiveness of Catheter Ablation in Left Ventricular Ejection Fraction, Stroke, Quality of Life, All-Cause Mortality, Sinus Rhythm Maintenance, and Hospitalization Rates as Compared to Medical Therapy

**DOI:** 10.7759/cureus.43372

**Published:** 2023-08-12

**Authors:** Mrinal J P Oble, Shamsun Nahar Sonia, Sherie George, Srushti R Shahi, Zahra Ali, Abdelrahman Abaza, Aneeque Jamil, Sai Dheeraj Gutlapalli, Marya Ali, Jihan Mostafa

**Affiliations:** 1 Internal Medicine, California Institute of Behavioral Neurosciences & Psychology, Fairfield, USA; 2 General Medicine, Pinderfields Hospital, Wakefield, GBR; 3 School of Medicine, California Institute of Behavioral Neurosciences & Psychology, Fairfield, USA; 4 Medicine, St. Martinus University Faculty of Medicine (SMUFOM), Willemstad, CUW; 5 School of Medicine, Bolan Medical College, Quetta, PAK; 6 Pathology, California Institute of Behavioral Neurosciences & Psychology, Fairfield, California, USA; 7 Internal Medicine/Clinical Research, California Institute of Behavioral Neurosciences & Psychology, Fairfield, USA; 8 Internal Medicine, Richmond University Medical Center Affiliated with Mount Sinai Health System and Icahn School of Medicine at Mount Sinai, Staten Island, USA; 9 Psychiatry, California Institute of Behavioral Neurosciences & Psychology, Fairfield, USA; 10 School of Medicine, Nishtar Medical University, Multan, PAK

**Keywords:** lvef (left ventricular ejection fraction), atrial remodeling, all-cause mortality, anti-arrhythmic drugs, hospitalization, medical therapy, heart failure, catheter ablation, atrial fibrilation

## Abstract

Atrial fibrillation (AF) in the setting of heart failure (HF) accounts for a significant proportion of mortality. AF can be managed either with rate control or rhythm control strategies. Rate control involves the use of beta blockers or calcium channel blockers. Rhythm control methods use antiarrhythmic drugs or catheter ablation (CA) to abolish the rhythm. Articles from PubMed and Google Scholar were chosen for review. The literature was reviewed for data from the last 10 years to be chosen for interpretation. Clinical trials, meta-analyses, and systematic analysis were included in this study. Various health parameters such as all-cause mortality, hospitalization rates, sinus rhythm (SR) maintenance, quality of life improvement, stroke risk, left ventricular ejection fraction (LVEF) improvement, and healthcare costs were analyzed. We demonstrated that CA was superior to medical therapy in reducing all-cause mortality and hospitalization. It leads to significant improvement in LVEF as SR was maintained consistently. Overall, quality of life improved in those who underwent ablation as compared to those who did not. Stroke risk reduction was seen in observational studies only. We recommend CA as first-line therapy for treating patients with AF in the setting of HF. More clinical trials are needed to determine the effectiveness of ablation in reducing stroke risk.

## Introduction and background

**Figure 1:**
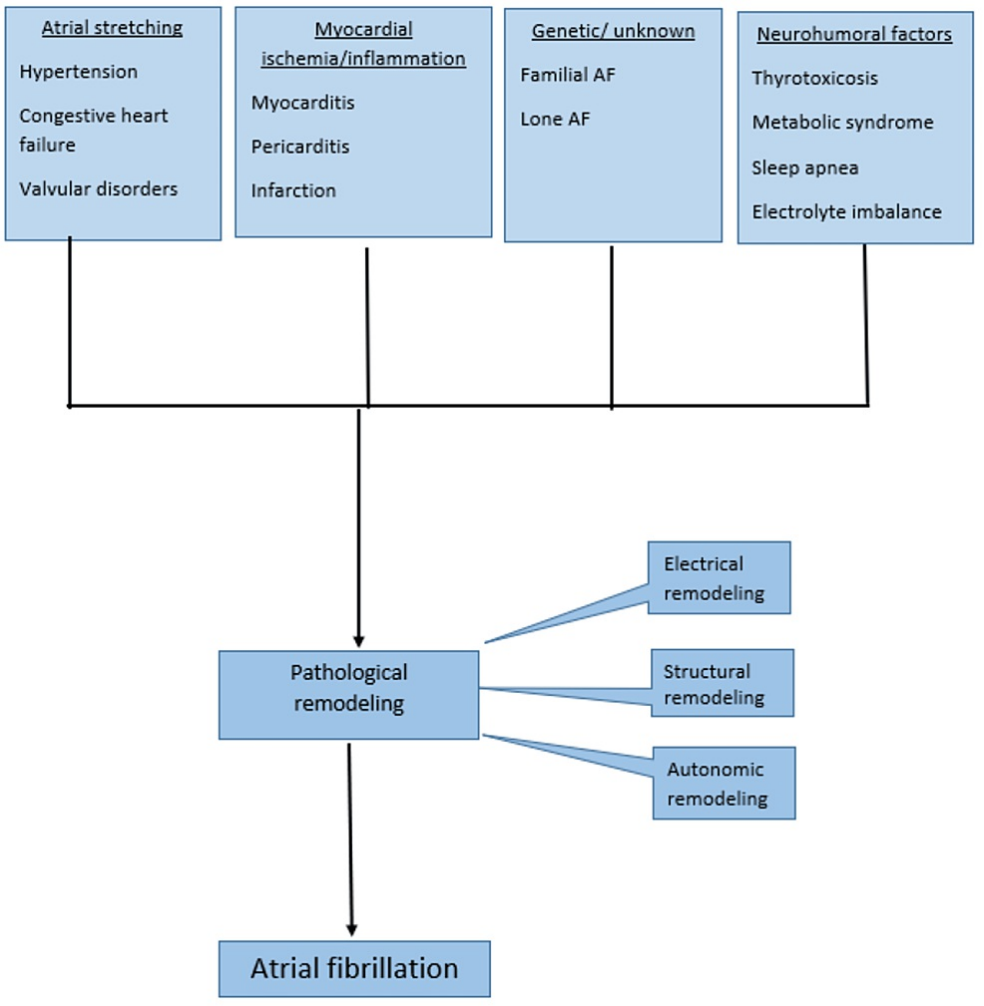
Atrial fibrillation pathophysiology AF: Atrial fibrillation Data from the study by Maruyama et al., (2012) [8] This image is a creation of the authors

Atrial fibrillation (AF) is characterized by disorganized, rapid, and irregular atrial activation with loss of atrial contraction and an irregular ventricular rate that is determined by atrioventricular (AV) nodal conduction [1]. Heart failure (HF) is defined as the abnormality of cardiac structure and/or function resulting in clinical symptoms (e.g., dyspnea, fatigue) and signs (e.g., edema, pulmonary crackles), hospitalizations, poor quality of life (QoL), and shortened survival [2]. Catheter ablation (CA) is a procedure where thin flexible tubes called catheters are inserted into veins or arteries to correct arrhythmias. It uses heat (radiofrequency) or cold (cryoablation) energy to create scars in the heart tissue to block the generation and conduction of the arrhythmias [3]. In the United States, approximately 454,000 hospitalizations with AF as the major diagnosis occur each year [4]. Every year, the illness kills approximately 158,000 people [5]. AF is also one of the top five causes of cardiac hospitalization, others being coronary atherosclerosis, heart failure, myocardial infarction, and stroke [6]. AF occurs due to pathological remodeling of the atria; it can be lone-standing AF or due to secondary to other heart diseases such as heart failure. Remodeling in AF can be grouped into three categories that include: (i) electrical remodeling, which includes modulation of L-type calcium current, various potassium currents, and gap junction function; (ii) structural remodeling, which includes changes in tissue properties, size, and ultrastructure; and (iii) autonomic remodeling, including altered sympathovagal activity and hyperinnervation [7]. This process is summarized in Figure 1.

**Figure 2:**
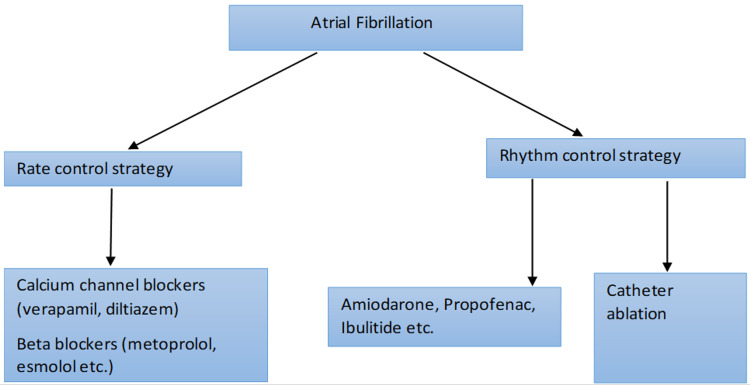
Management of atrial fibrillation Data from the study by Amin et al., (2016) [9] This image is a creation of the authors

AF and HF are usually present together, and therefore, management of AF in HF is important to reduce mortality and morbidity in patients. Currently, A-fib can be managed with either medical therapy (MT) (rate control) or by catheter ablation (rhythm control) of the arrhythmia [9]. These methods of management are explained in Figure 2 in further detail. CA is an invasive procedure that requires sedation or general anesthesia during the procedure. This traditional review tries to establish the effectiveness of ablation vs. pharmacological therapy in terms of mortality and morbidity benefit to patients. It tries to understand the factors that determine the kind of treatment a patient may choose, like the adverse effects associated, availability and access to treatment options, co-morbidities, sex, cost of treatment, etc. Determining the effectiveness of either type of management over the other can help us reduce the burden on healthcare services, ensure patient compliance with therapy, reduce the duration of therapy, and improve access to healthcare services.

## Review

Method

**Figure 3:**
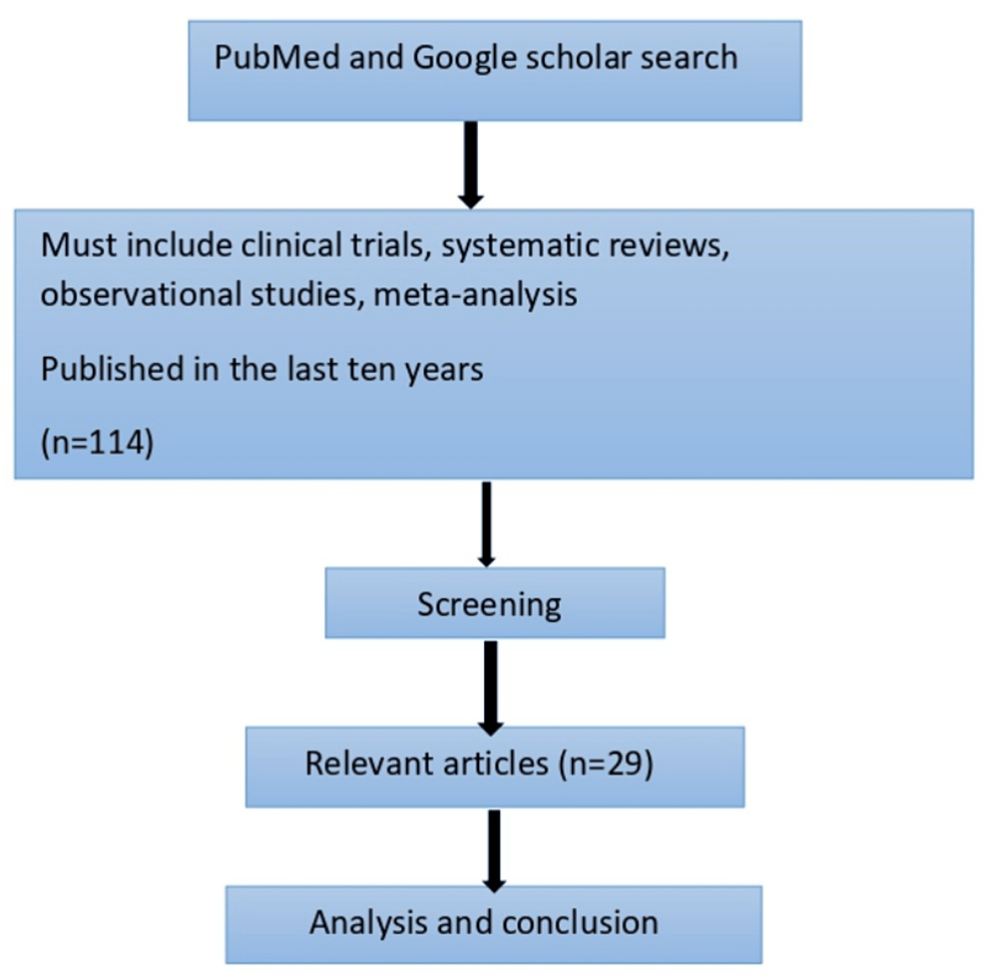
Selection and filtering data

This literature review was conducted by looking up articles on two databases, namely, PubMed and Google Scholar. We included clinical trials, systematic reviews, observational studies, and meta-analysis that were published in the last 10 years. Initial search yielded 114 studies. After carefully reviewing each study for relevant data, we selected 29 studies to include in our review. The method is summarized in Figure 3.

Discussion

AF and HF are common heart conditions that are associated with substantial morbidity and mortality. These diseases often coexist [10-14]. Over the next two decades, these two disorders are expected to increase in their prevalence [15]. Despite remarkable improvement in MT, HF remains an important public health issue and is associated with substantial healthcare costs. Patients with AF and HF have even higher mortality and hospitalization irrespective of presentation, although AF usually develops following longstanding HF due to dilation of the atria. AF can also occur due to different pathophysiologic changes like left ventricle (LV) dysfunction due to loss of atrioventricular synchrony and tachycardia-induced cardiomyopathy [16,17]. Atrial structural remodeling usually occurs in HF due to the sustained increase in circulatory volume and tension and neurohormonal stress [18], increasing the chances of development of AF. Irrespective of the type of AF or degree of HF, the combined presentation of the diseases is known to adversely affect the patients’ prognosis, causing a massive burden on the healthcare system, and reinforcing the need for developing efficient therapies in this patient demographic [15].

CA of the pulmonary vein sinus is performed as the ectopic firings from the pulmonary veins initiate AF in the majority of the patients. With improvements in catheter design and visualization technology, CA as a management strategy for AF aiming at rhythm control has been increasingly used nowadays [15,19].

LVEF Improvement

HF was classified based on the New York Heart Association (NYHA) [20] and American College of Cardiology guidelines [21]. The main findings of this analysis showed that patients with HF and LVEF < 50% treated with CA had significant clinical and echocardiographic improvement at 12-month follow-up and a higher life expectancy. Patients with concomitant AF and HF with reduced LVEF have an increased mortality rate and morbidity, including stroke, worsening HF, sudden death, and reduced quality of life [22-24]. All-cause mortality is significantly higher in AF patients with heart failure with reduced ejection fraction (HFrEF) compared with heart failure with preserved ejection fraction (HFpEF). AF promotes the clinical deterioration of patients with preexisting HF as atrial contraction significantly contributes to ventricular filling [16]; moreover, AF can worsen ventricular function by inducing tachycardiomyopathy [25,26]. LVEF change was shown to improve in patients who underwent CA at the one-year follow-up as compared to those who did not receive any therapy [25,27]. LVEF improvement was also significantly better in patients who underwent CA as compared to rate control therapy [27].

The randomized controlled trial (RCT) conducted by Marrouche and his colleagues of CA vs MT for patients in HF shows some benefit that leans toward CA. Further research is needed for large-scale adoption from this trial [28]. A reduction in hospitalization due to HF in those who underwent CA was noted, but the mortality benefit was not seen for at least three years after the CA. Those with a higher ejection fraction (above 25%) seemed to have a better outcome compared to those with a low ejection fraction (below 25%). Although CA showed significant benefits, it did not lead to complete elimination of AF in the patient population; that is, the recurrence of AF was common. The trial also demonstrates that CA as compared to MT has a better chance of maintaining sinus rhythm (SR) [29-31]. After five years of the study, 63% and 22% of patients in the CA and MT groups, respectively, maintained SR [31]. Furthermore, the mean AF burden during the study ranged from 20% to 27% in the CA group compared with 48% to 64% in the MT group. This trial demonstrates the evidence of the benefit of CA vs MT in clinical outcomes like hospitalization rates, reduction in disease burden, left ventricular ejection fraction improvement, and QoL in patients with HF [31-33].

Atria Remodeling

In patients undergoing CA, a reduction in the left atria (LA) diameter and a subsequent improvement in LVEF were observed in the study by Lee et al. [22]. The mechanism of LA dysfunction changes based on the degree of HF. HFpEF is mostly caused by abnormalities in excitation-contraction coupling and ventricular stiffness and is usually associated with diastolic dysfunction. In contrast, HFrEF is more likely due to ischemic etiology and left bundle branch block. The HF with mid-range ejection fraction (HFmrEF) cohort is closer to the HFrEF group with respect to age, sex, systolic blood pressure, and ischemic etiology but differs in the degree of cardiac fibrosis [34]. It was demonstrated in the study by Lee et al. that AF can cause fibrosis and the severity of the cardiac fibrosis is directly proportional to the AF burden. This observation leads us to infer that CA can help maintain heart function in this subset of patients. In another study, it was observed that remodeling changes can only be reversed by CA [25].

Hospitalization

In a UK study on the one-year follow-up mark, AF patients treated with MT had more than twice the average number of heart-related outpatient visits as compared to the CA population [35]. A reduction in mortality observed in the CA cohort in our study demonstrates the potential health benefit associated with CA as compared to AAD therapy [36,37]. Similar to this study, Jarman et al. (2017) observed a lower likelihood of mortality among AF patients who underwent CA as compared to the untreated group [35]. According to the study by Lima et al., the 30-day readmission rates were lower for patients treated with CA compared to MT, while patients receiving the latter were sicker [38]. The study also observed a 14% lower risk for 30-day readmission in the CA cohort when compared to the MT cohort. Among the reasons for readmission, HF and arrhythmias accounted for the majority of the causes. The service providers of Medicare and Medicaid have used the 30-day readmission rates as indicators of hospital performance. Long et al. also demonstrated the benefit of CA in reducing hospitalization rates among patients [39].

Stroke Risk

Based on the EASTAFNET4 trial, the risk of stroke shows a non-statistically significant reduction in those who undergo CA [40]. Multiple studies showed significant improvements in cardiac function, HF hospitalizations, mortality, and stroke incidence [39]. It was also of note that the benefit in stroke reduction was observed mainly in observational studies and the benefit from the RCT was not evident [41,42]. The benefit of stroke reduction observed in some studies seems to be lost due to periprocedural event risk [42].

In the CASTLE-AF study, the number of episodes of ischemic stroke was too small and underpowered to demonstrate the benefit of CA in stroke prevention. Previous studies have shown a reduction in stroke occurrence in AF patients. However, CA performed in the patients was not in the setting of HF [43]. According to Zeng et al., there was no significant difference between CA and antiarrhythmic drug (AAD) therapy in the risk of the occurrence of stroke; however, it was observed that this finding was due to publication bias. Upon re-analyzing and correcting the data using the trimming and filing method the findings remained consistent [44]. To summarize, more RCTs are needed to establish the benefit of ablation in stroke reduction.

Adverse Effects of CA

CA had many procedure-related complications such as tamponade, femoral atrioventricular fistula, pseudoaneurysm, major bleeding, stroke, gastroplegia, hemoptysis, and pulmonary vein stenosis [28,45]. Non-procedural adverse effects include hyperthyroidism, hypothyroidism, symptomatic bradycardia, and tachycardia-bradycardia syndrome [46]. It was observed that CA-related adverse effects were significantly higher in HF populations compared to the general AF population. However, the reduction in mortality, LVEF improvement, and reduced hospitalization were consistent across the study groups [43]. The adverse effects are compiled in Table 1.

**Table 1:** Adverse effects related to ablation Data from the study by Marrouche et al. [28], Anselmino et al. [45], Wu et al. [46].

Procedure related	Non-procedural
Cardiac tamponade	Hyperthyroidism
Femoral, atrioventricular fistula	Hypothyroidism
Pseudoaneurysm	Symptomatic bradycardia
Major bleeding/ hemoptysis	Tachycardia-bradycardia syndrome
Stroke	
Pulmonary vein stenosis	

CA, as a first-line approach, might be particularly important for HF patients, since arrhythmic recurrences promote worsening cardiomyopathy, arrhythmia progression, and poor outcomes [10].

SR Maintenance

Multiple studies have demonstrated superior efficacy in terms of reduction of AF recurrence and symptoms with CA over AADs [18,29,30,35,47-49]. Based on the EASTAFNET4 trial, CA is more effective than AADs in preventing recurrent AF [40]. In another trial, it was observed that SR maintenance was essential for preventing mortality [16,50]. The superiority of CA versus AADs has been demonstrated in the randomized AATAC trial, in which HFrEF patients with persistent AF receiving CA were significantly more likely to be free from AF recurrence (70% vs. 34%, P < 0.001) and have lower mortality rate (8% vs. 18%) than the patient receiving amiodarone [22,49,51].

A study by Long et al. demonstrated that CA in AF patients achieved higher rates of SR. A randomized study showed that CA in AF patients with HF (70%) is superior to amiodarone (34%) in maintaining SR at the 24-month follow-up [39]. It was observed that SR maintenance had to occur for LV function to improve [38]. Similar findings were reported in other studies [10,52,53]. Numerous factors contributed to the SR maintenance, like age; gender; AF types; LVEF value; etiology of HF; duration of AF and HF; and different CA techniques, procedures, and physician experience. It is therefore essential that management is guided toward therapies that maintain SR.

Cost of Treatment

In a UK study to establish the cost-effectiveness of CA treatment as compared to AAD treatment among AF patients, it was found that the cost of treatment (Table 2) of CA cohort to MT cohort came up to £12,500 and £15,300 per quality-adjusted life-year (QALY) [35]. As healthcare resources are limited, management using CA for AF could offer patients significant economic benefits as compared to conventional drug treatment [35]. The study by Leung et al. demonstrated that CA was a highly cost‐effective strategy for patients suffering from AF as compared to MT. Even though CA had a higher up‐front cost it had a highly significant decrease in cardiovascular adverse events and AF recurrence during follow‐up which led to reduction in healthcare-related expenditure over the patient’s lifetime [36].

**Table 2:** Cost of treatment QALY: Quality-adjusted life years Data from the study by Jarman et al. [35]

Medical therapy cost per QALY	Catheter ablation cost per QALY
£12,500 ($15,630)	£15,300 ($19,131)

The National Institute of Clinical Excellence (NICE) in 2021 published the results of their cost‐effectiveness analysis comparing AADs to different types of CA techniques including radiofrequency (RF) with point-by-point (PP) and cryoballoon CA over a lifetime duration. Among these methods, RF and PP CA was found to be the most cost‐effective option [36]. The study by Lima et al. demonstrates that CA has higher index hospitalization costs, but lower readmission costs which is beneficial to patients over a lifetime [38].

All-Cause Mortality

Lee et al. (2023) demonstrated the substantial benefit of CA in improving all-cause mortality and HF hospitalization rates in HF patients with paroxysmal or persistent AF. But in the AMICA trial, the same benefit was not observed in all patients with AF and HF [22]. The study also demonstrated that the benefit of CA is greater in patients with relatively better LVEF and less advanced HF stage [31]. This observation was also supported by Fujimoto et al. who observed that compared to HFrEF, patients with HFpEF had lower all-cause mortality, HF hospitalization, and stroke or systemic embolism [54]. It is important to note that according to Lee et. al. all-cause mortality was reduced in all the subgroups irrespective of the degree of HF [22]. The meta-analysis conducted by Magnocavallo et al. demonstrated similar findings that CA was associated with a 25% reduction in all-cause mortality and hospitalization compared to MT [10].

The study by Lima et al has shown AF to be an independent predictor of death and HF readmission among patients with HFpEF [38]. Although previous clinical trials have excluded HFpEF from the study groups, recent observational data show that CA may provide a clinical benefit in these patients. In the intention-to-treat analysis of the CABNA trial, no difference was noted in mortality and hospitalization rates, but this was most likely due to large crossover and lower than expected mortality rates [41].

Comparing the clinical benefit of CA to drug therapy in racial and ethnic minorities in the CABNA trial found significant reductions in the combined primary endpoint of death, stroke, serious bleeding, or cardiac arrest (68% relative risk reduction), all-cause mortality (72% relative risk reduction), and time to first recurrence of AF (55% relative risk reduction). Adverse events in both treatment cohorts were low and showed no difference between racial and ethnic minorities and nonminorities [55].

Quality of Life Improvement

In a UK-based study, it was noted that the quality-adjusted life expectancy was between 11.75 and 12.20 years for CA and 11.00 to 11.35 years for the AAD group [35]. Several randomized trials conducted in Europe identified a beneficial effect of rhythm control therapy using CA in participants with AF and HF with reduced ejection fraction [16,40]. The EASTAFNET4 trial identified a 21% reduction in all-cause mortality from cardiovascular causes, stroke, or hospitalization with worsening of HF in participants receiving rhythm control within one year of diagnosis compared with those who received conventional MT [40]. The clinical benefit of early initiation of rhythm control therapy was observed both in asymptomatic and symptomatic participants.

The ARC-HF and CAMERA-MRI trials demonstrate that rhythm control by CA is superior to rate control in improving quality of life, and LVEF improvement [22]. Although both AADs and CA led to burden reduction of AF, patients who underwent CA had better outcomes such as improved all-cause mortality and reduction in hospitalization [22]. An investigation by Barra et al. noted that in both RCTs and observational studies, a survival benefit of CA was observed, but the evidence was noted in studies performed specifically in the HF, concluding that more research is needed to make the findings generalizable to the general AF population [42]. Overall, CA led to significant improvements in QoL and is preferred as a valid therapy option.

All the parameters discussed in this study and the changes observed due to CA are summed up in Table 3.

**Table 3:** Health parameters analyzed in the study LVEF: Left ventricular ejection fraction; QoL: quality of life Data from the study by Magnocavallo et al. [10], Lee et al. [22], Cirasa et al. [25], Chen et al. [27], Marrouche et al. [28], Mont et al. [29], Briceño et al. [30], Greet et al. [31], Jarman et al. [35], Leung et al. [36], Saglietto et al. [41], Barra et al. [42], Packer et al. [49], Machino-Ohtsuka et al. [51].

Parameters	Change	P values
All-cause mortality	decreases	<0.05
Hospitalization rates	decreases	<0.05
LVEF	increases	<0.05
QoL	increases	<0.05
Stroke risk	Decreases (only observational studies)	>0.05 Not significant
Cost of treatment	lower	<0.05

Limitations

This study has used data from all types of articles, systematic reviews, meta-analysis, and clinical trials only from the last 10 years. Also this review is a hypothesizing and post hoc analysis of the various parameters that indicate the health of patients suffering from AF in the context of HF. Since we used secondary database for this study, misclassifications, underreporting, and missed diagnosis could have had an impact on the results.

Summary and Future Prospects

To summarize, this study tries to determine the changes observed in various parameters such as all-cause mortality, hospitalization rates, LVEF, QoL, atrial remodeling, sinus rhythm maintenance, stroke risk, and cost of treatment after CA as compared to pharmacological therapy. Most of the parameters show a statistically significant positive effect upon CA. Stroke risk changes also show reduction but was not statistically significant and the reduction in risk noticed was only seen in observational studies. To fix this grey area, we recommend more RCTs specifically designed to study the changes in stroke risk following CA.

## Conclusions

In summary, this review compared the effectiveness of catheter ablation to medical therapy. It was demonstrated that CA was superior to MT in improving LV function, Sinus rhythm maintenance, all-cause mortality, and QoL. Reduction in stroke risk was only seen in observational studies but ablation is recommended in all patients to reduce AF burden. In this study, we break down various parameters of morbidity associated with AF in the setting of HF and try to compare each parameter and draw a conclusion. We recommend more RCTs to determine the efficacy of ablation in reducing the risk of ischemic stroke as the results in the previous studies were noncontributory.
